# Characterizing Overall Survival of Patients with Acute Myeloid Leukemia: A Competing Risk Analysis of SEER Data Covering 46 Years †

**DOI:** 10.3390/cancers17233735

**Published:** 2025-11-22

**Authors:** Dennis Görlich, Leonas Lanwer, Cristina Sauerland, Utz Krug, Andreas Faldum

**Affiliations:** 1 Institute of Biostatistics and Clinical Research, University of Münster, 48149 Münster, Germany; 2 Faculty 3 Mathematics and Computer Science, University of Bremen, 28359 Bremen, Germany; 3 DKMS Collection Center, 50670 Cologne, Germany

**Keywords:** acute myeloid leukemia, overall survival, competing risk analysis, historic perspective

## Abstract

Background and Methods: We analyzed trends in overall survival after acute myeloid leukemia (AML) using US registry data covering the years 1975 to 2021. Our study utilized unique patient data from the Surveillance, Epidemiology, and End Results Program (SEER), with 37,615 adult patients included. We employed Kaplan–Meier estimates and competing risk analyses to assess overall survival and risks of AML-related death and death from other causes. Results: Our findings indicate a clear and continuous trend of improved survival in AML patients, attributed to decreased AML-related mortality. Patients aged 60 or younger showed continuous improvement over the entire period, while those aged 61 or older demonstrated improvement starting in the early 2000s. Only patients receiving chemotherapy treatment benefited from ongoing research. No sex-specific differences were found. Subgroup analyses revealed age-dependent differences in survival improvement. The delayed onset of improvement in older patients may be due to the availability of hypomethylating agents and increased access to hematological stem cell transplantation. Conclusion: Our analysis showed, on a high level of abstraction, how ongoing research and availability of new treatment options improved survival for AML patients. Furthermore, subgroup analyses revealed age-dependent differences in this respect. While the data did not allow us to conduct in-depth analyses of treatment regimens, we hypothesized that the later onset of a pattern of improvement in older patients was due to the availability of hypomethylating substances and increased availability of hematological stem cells transplantation for elderly patients.

## Simple Summary

Acute myeloid leukemia (AML) is the most abundant leukemia in adults and, if being untreated, results in death. Available therapies for AML patients have changed drastically during the last decades. Newly available, more effective, therapies increase survival rates. We analyzed US cancer data collected in the Surveillance, Epidemiology, and End Results (SEER) registry to characterize the improvement in survival in more detail. To separate treatment related effects from other influences, we employed a competing risk survival analysis. Our analysis of more than 37,000 AML patients showed that the continuous yearly improvement of patient survival after AML exists, but in the older patient subgroup this trend started later than for younger patients. The analysis of the “competing event” (non-AML death) showed no progress over time. Overall, using a hazard ratio based approach combined with competing risk analysis, we demonstrated the impact of cancer research and changes in the health system on AML patient survival.

## 1. Introduction

Acute Myeloid Leukemia (AML) represents a heterogeneous group of hematological malignancies in the myeloid line characterized by rapid disease progression and poor prognosis. Despite significant advancements in the understanding and treatment of AML, life expectation, in terms of the observed overall survival (OS) times, remains low for many patients. For the USA, the Surveillance, Epidemiology, and End Results (SEER) estimated a five-year relative survival rate of 31.9% and reports 20,800 new cases in 2024.

Over the past several decades, the treatment landscape for AML has undergone substantial evolution, driven by advances in research and clinical practice [1,2,3,4]. Recent advancements in the understanding of the genetic and molecular underpinnings of AML have facilitated the development of targeted therapies, which have shown promising results in improving response rates and survival outcomes for molecularly defined patient subgroups [5]. Furthermore, the emergence of immunotherapies, including monoclonal antibodies and cellular therapies, represents a significant shift toward more personalized and less toxic treatment strategies [6,7]. Previous studies analyzing historic trends in OS reported improvement by means of relative survival estimates [8,9]. Most recently, a study by Vener et al. presented a large comprehensive survival pan-European analysis of long-term survival of myeloid leukemias among 277,000+ cases, diagnosed between 2001 and 2014 [10]. Similarly, also based on SEER data, Sasaki et al. presented an analysis of survival trends between 1980 and 2017 based on Kaplan–Meier estimates [11].

Our investigation utilizes the comprehensive SEER dataset (Surveillance and End Results (SEER) Program, www.seer.cancer.gov, accessed on 1 November 2024, National Cancer Institute, DCCPS, Surveillance Research Program; 2024) to conduct a competing risk analysis concerning the survival of patients with AML over a span of 46 years. The objective was the elucidation of temporal trends in overall survival and alignment of these trends with the evolution of treatment and management strategies for AML. While competing risk analysis on SEER data are not uncommon [12,13,14,15], our study, for the first time, employed the Fine and Gray model [16] for a competing risk analysis (CRA) to derive annual hazard ratios (HR) against a uniform reference point, to analyze AML survival data. CRA are routinely employed for analysis tasks, where more than one event (such as death, relapse, or transplantation) is in contention to occur next in a patient’s clinical trajectory. In the subsequent sections, we delineate our methodology and present the outcomes of our analysis regarding historical trends in AML survival.

This article is a revised and expanded version of preliminary work entitled “Characterizing overall survival in AML patients: A competing risk analysis of SEER data covering 46 years”, which was presented at the International Symposium on Acute Leukemias (ISAL2025, Munich, Germany, March 2025) [17].

## 2. Materials and Methods

This study conducted a comprehensive CRA to assess survival in patients diagnosed with AML, using data from the SEER program, spanning years 1975 to 2021. The SEER program collects cancer incidence and survival data from population-based cancer registries covering approximately 46% of the U.S. population. Data was accessed under the SEER researcher data use agreement (to DG).

### 2.1. Study Population

Our study included adult patients with a confirmed diagnosis of AML made between 1975 and 2021. AML patients were identified using the International Classification of Diseases for Oncology (see Supplementary Table S1 for included ICD-O-3 codes). We included individual patient data with complete data on demographics (age, sex, race/ethnicity), diagnosis date, and follow-up status. Patients with missing critical information, as well as duplicates, were excluded from the analysis. Patients were censored in our analyses when alive at the last follow-up. Supplementary Table S2 gives an overview on the follow-up status with respect to complete date information of included patients as coded by SEER.

### 2.2. Data Extraction and Variables

Key demographic variables extracted from the SEER database included age at diagnosis, sex, race/ethnicity, year of diagnosis, clinical characteristics, chemotherapeutic treatment, cause of death (COD) and time to death or end of observation. Complete citation of the SEER data source was given in Supplementary Text S1 including the list of extracted variables. With respect to the CRA, information regarding COD was classified either to AML-related mortality (as indicated in the COD variable) or death from other causes. Other than AML COD were re-classified for a subsequent analysis into broader categories, i.e., cardiovascular diseases, haematologic cancers, infections and sepsis, non-cancer condition, other solid cancers, and other COD (including, e.g., “Accidents and Adverse Effects” and “Suicide”). Details on the redefined classes for COD were reported in Supplementary Table S3.

The primary outcome of interest was overall survival (OS), defined as the time (in months) from AML diagnosis to death from any cause. Patients were censored when alive at the last follow-up. Furthermore, we performed a competing risk analysis described below.

### 2.3. Statistical Analysis

Kaplan–Meier (KM) survival estimates [18] were initially computed to visualize differences across decades. We extracted early death (ED) rates as 1-month and 2-month KM estimates from this analysis. To account for competing risks, we utilized the Fine-Gray subdistribution hazard model [16]. The subdistribution hazard has a direct relationship with the cumulative incidence function. Specifically, “the regression coefficients from a Fine-Gray subdistribution hazard model can be indirectly interpreted as the regression coefficients for a complementary log-log generalized linear model for the CIF similarly to hazard ratios without competing risks.” (cp. [16,19,20]). Additionally, following the justification of Lau et al. [21] subdistribution models are better suited in analysis contexts which aim at incidence (or prediction, which we did not intend). This interpretation was also taken by Austin, Lee, and Fine [22], as well as Wolbers at al. [23].

Temporal trends in survival were examined by computing HRs for each year from 1976 onwards, using 1975 as the reference for all other years. Technically, year of diagnosis was modeled as a fixed factor. The proportionality assumption was assessed visually in log(-log)-plots of the cumulative incidence function and time. The inspection showed no severe violations of proportionality. For more technical information see Supplementary Text S2.

Primarily, we fitted Fine and Gray models for the competing events AML-related death and the composite event consisting of all other non-AML COD, as our main analysis of temporal trends. Subsequently, separate CRAs were performed for each category of non-AML COD, considering all other events as competing events. For all analyses, patients alive at last follow-up were censored. Consistently, all hazard ratios estimated by the Fine-Gray model were reported as sHR, while estimates of OS were reported as HR. Yearly sHRs and 95%CI were reported. A LOESS fit was used to illustrate trends in HRs over time. Covariates were analyzed in the same manner for the main analysis, by modeling an interaction term between year of diagnosis and the respective covariate (age group, sex, therapy). As accompanying analysis, cumulative 12-month incidences (95%CI) for COD were estimated and Gray’s test applied to detect differences between decades. As sensitivity analyses, classical Cox-proportional hazard model (Cox-PH) were fitted to obtain yearly HRs for each year from 1976 onwards, using 1975 as the reference for all other years. Also, an adjusted analysis, by using age and sex as fixed cofactors in the Fine and Gray model, was estimated. Sensitivity analyses were reported in Supplementary Figure S1. The data analysis for this paper was generated using SAS software (Version 9.4, Copyright © 2024 SAS Institute Inc. SAS and all other SAS Institute Inc. product or service names are registered trademarks or trademarks of SAS Institute Inc., Cary, NC, USA).

### 2.4. Ethical Considerations and Data Sharing

This study utilized de-identified data from the SEER database, which is available for research purposes under a research license. Consequently, this research did not require institutional review board approval. No original data can be shared by the authors, due to the granted research license. Aggregated estimates were provided in a data table as Supplementary Table S4.

## 3. Results

### 3.1. Description of the Study Population

**Figure 1:**
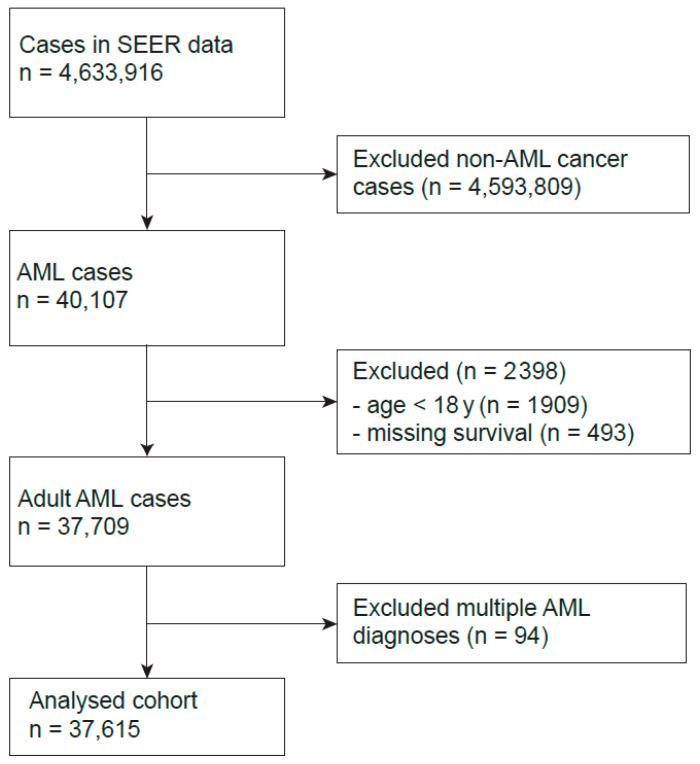
Flow chart describing the data filtering steps to generate the analyzed AML cohort.

In total, 4,633,916 cancer cases have been extracted from the SEER registry. Thereof, only adult AML cases have been filtered and included into our analysis. When a patient experienced more than one episode of AML, only the first occurrence has been kept for analysis, leading to a final sample size of 37,615 (Figure 1). Descriptive statistics of the available set of covariables (sex, age at diagnosis, chemotherapy, diagnostic confirmation, first tumor, and race) have been reported in Table 1. Sample sizes for each year of diagnosis were given in Supplementary Table S5. Noticeably, the sex distribution was slightly skewed towards females, who accounted for 54.6% of the cohort. The ethnical composition highlights a predominance of white patients, accounting for 84.0% of the analysis set, with black and other ethnic groups constituting 5.9% and 9.9%, respectively. Ethnicity was unknown for only 0.3%. The majority, 67.0%, of patients received some form of chemotherapy, underscoring its role as a primary treatment modality.

### 3.2. Historic Changes in Cofactors

We observed some noticeable developments in the composition of relevant cofactors over the last decades. Since these might affect the interpretation of results, we described, here, changes in the distributions of sex, chemotherapy availability, inclusion on AMLs as non-primary tumors, and changes in ethnicity. Supplementary Tables S6 and S7 presented the distribution of available cofactors by decade. It showed, that, except the sex distribution, all other variables changed over time. The patient’s mean age at diagnosis increased from 63.1 years in the 1970s to 67.4 years in the 2020s (*p* < 0.0001). The percentage of patients who received chemotherapy increased from 61.1% to 73.2% (*p* < 0.0001) between the 1970s and 2020s (please note that the treatment variables can be severely biased in the SEER dataset). Also, the number of patients who acquired their AML as first malignant disease reduced over time, while vice versa more patients were included into the data who had other cancer diseases before AML (*p* < 0.0001). In clinical trials, these patients would very likely be classified as secondary or treatment-related AMLs depending on the pathological mechanism. Ethnicity distribution shifted (*p* < 0.0001) towards an increased ethnic diversity.

### 3.3. Overall Survival and Early Death Rate

**Figure 2:**
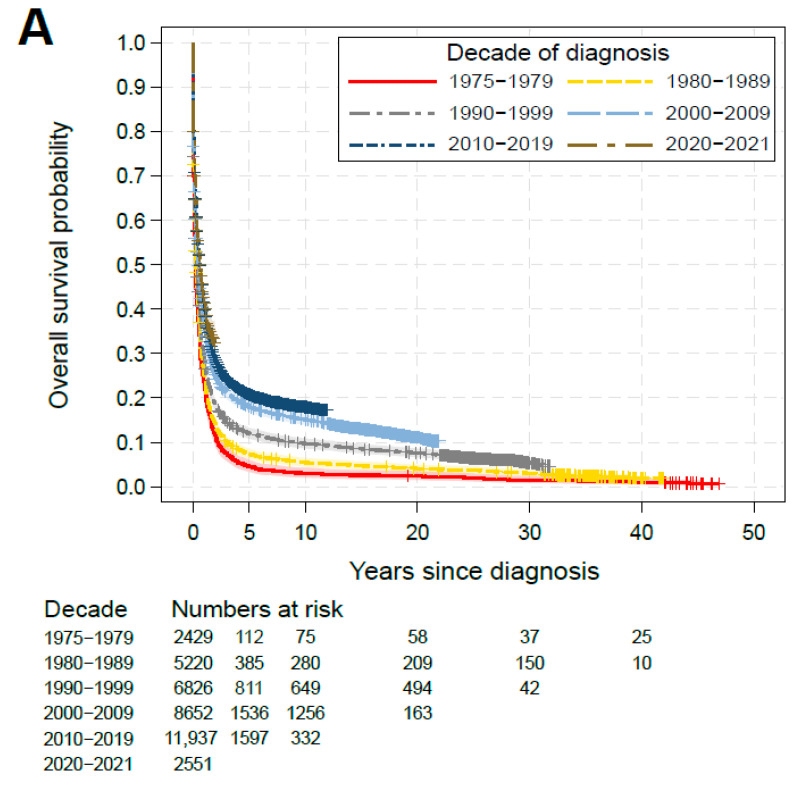

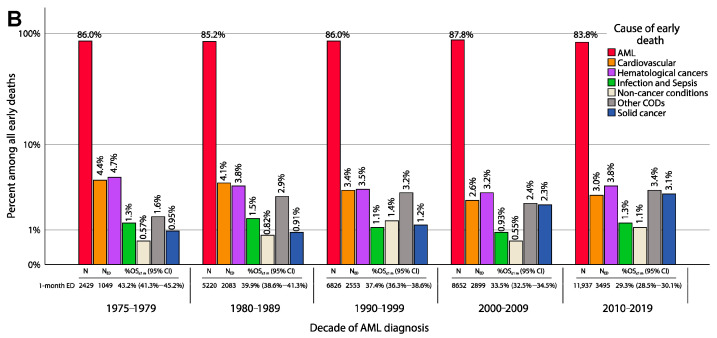
Overall survival analysis and early death. (**A**) Kaplan–Meier estimates by decade. (**B**) Distribution of causes of death after AML, within the early death subgroup (OS ≤ 1 month; 12,829 early deaths of in total 32,191 documented deaths). Statistics on early death probability by decade were given in the table below the bar chart.

The data showed a clear trend for increasing survival probabilities for AML patients over successive decades. In the 1970s the 5-year survival probability was 4.5%, while in the 2010s already 20.8% of patients were expected to survive at least five years after their AML diagnosis (Figure 2A, *p* < 0.0001). One-month early death (ED) occurred in around 43.2% (95%CI: 41.3% to 45.2%) of patients in the 1970s, while in the 2010s the rate dropped to ca. 30% (95%CI: 28.2% to 31.8%; *p* < 0.0001; details for all decades shown in Figure 2B). Two-month ED occurred in around 51.0% (95%CI: 49.0% to 53.0%) of patients in the 1970s, while in the 2010s the rate dropped to ca. 35.1% (95%CI: 34.2% to 35.9%; *p* < 0.0001, all decades shown in Supplementary Table S8). As shown in Figure 2B, the distribution of reasons for early death did not change dramatically when compared between decades. Though, the statistical test (Chi-Squared) indicated a shift towards more early deaths due to solid cancers among minor fluctuations among the other classes (*p* < 0.0001).

### 3.4. Competing Risk Analysis:

#### 3.4.1. AML Related Mortality

**Figure 3:**
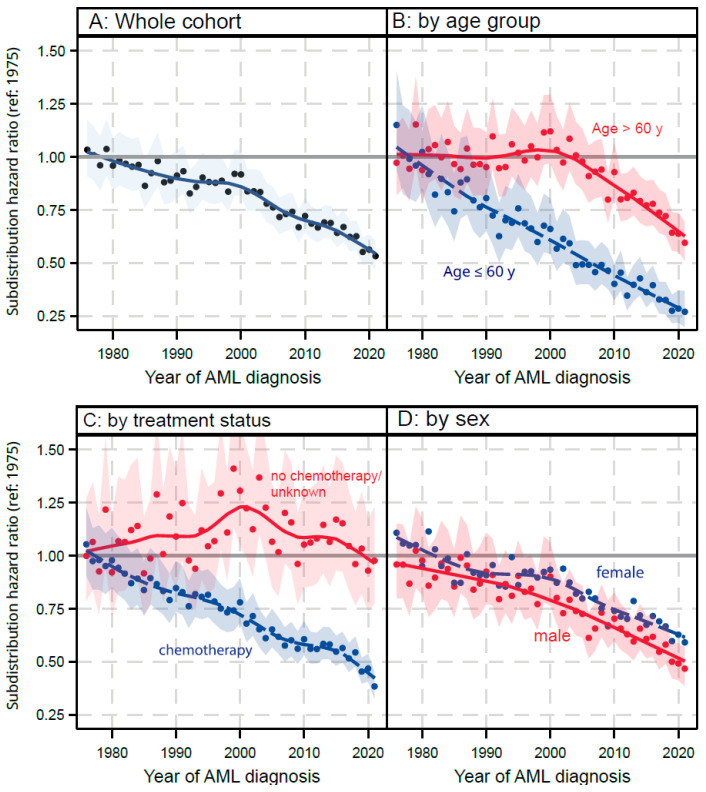
Yearly sub-distribution hazard ratios for the risk to die from AML. (**A**) whole cohort; (**B**) by age group (≤60 y vs. 61+ years); (**C**) by treatment status; (**D**) by sex. Solid lines show LOESS fits. Confidence bands show point-wise 95% CI from the competing risk model. Numerical values for each year are available in the Supplementary Table S4.

The analysis of sHRs for AML-related death, using 1975 as a baseline year, reveals significant trends in treatment outcomes over subsequent decades. Our CRA showed that, overall, there was a continuous improvement of survival since the 1970s in the pooled cohort of AML patients (Figure 3A). This effect maintains under adjustment for age and sex (sensitivity analysis, Figure S1). An analysis stratified by age (≤60 years vs. 61+ years of age at diagnosis, Figure 3B) showed a differential pattern. While younger patients benefited from a continuous improvement in survival rates, the older AML population stagnated until the early 2000s. Then, a trend of improvement set in at approximately the same rate of improvement as found in the younger subgroup. Age was a clear predictor of this pattern, while treatment (yes/no, Figure 3C) and sex (Figure 3D) did not contribute to the effect.

#### 3.4.2. Non-AML Reasons for Death

**Figure 4:**
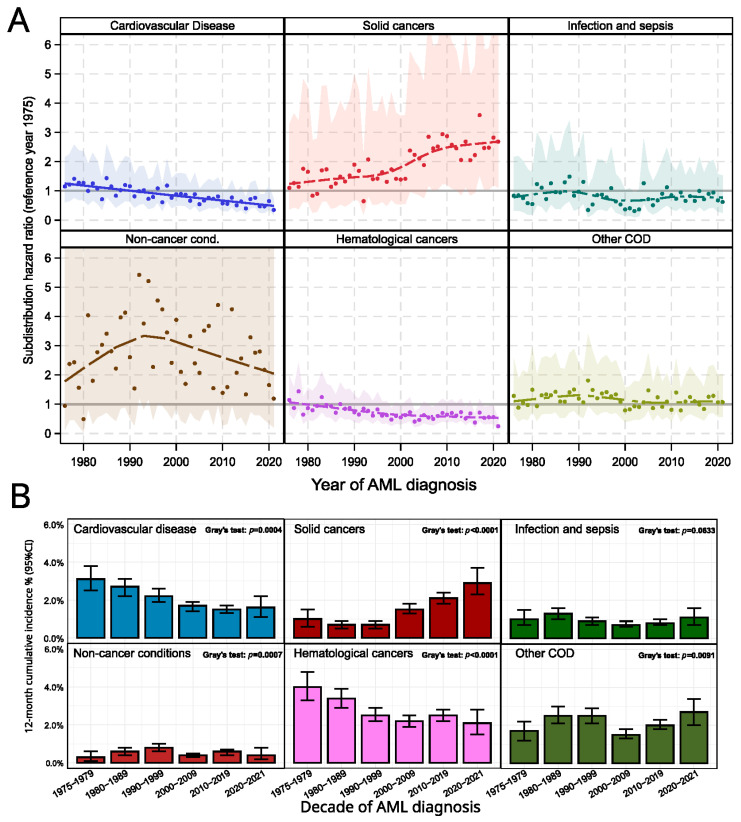
Panel (**A**) Yearly sub-distribution hazard ratios (sHR) and 95% CI for the defined classes of other causes of death (COD) other than AML. Common reference year was 1975. Panel (**B**) Bar charts show 12-month cumulative incidences and 95% CI for the respective events over decades, estimated from the Fine-Gray models.

The data overall showed changes in the distribution of causes of death other than AML over the decades. Absolute and relative frequencies were presented in Supplementary Table S9. Especially, cardiovascular deaths reduced from 4.6% to 2.9% (*p* < 0.0001, Chi-squared test against all other reasons including AML-related death) throughout the last decades (1970s to 2020s). Deaths due to solid cancers seemed to increase (1.6% to 4.8%, *p* < 0.0001). Other hematological (non-AML, non-myeloid) cancer diseases occurred after AML. Interestingly, the proportion among deaths after AML reduced from 5.2% to 3.3% during the last decades (*p* < 0.0001). No shift in rates was observed for infections (*p* = 0.1048) and other COD’s (*p* = 0.0502). For non-cancer conditions that were lethal, an intermittent increase was observed (from 0.7% to ca 1.5%) for the 1980s to 2010s (*p* = 0.0108). In future, the extended follow-up of the 2020s cohort will show whether this level is kept, or if the trend reverses. Figure 4A shows the yearly sHR for each of the competing other CODs. In Figure 4B, 12-month cumulative incidences per decade were given. The Gray-tests show noticeable differences in the cumulative incidence between the decades, consistent with the effects found in the sHR analysis. While the CRA estimates summarized whether the hazard changed between years, the absolute and relative frequencies (cp. Supplementary Table S9) changed due to increased OS, which leaves the chance that patients lived to a following terminal event, the CRA incorporates overall survival trends for these events. The analysis of annual HRs did not yield enough power to detect trends in the non-AML COD; the more coarse-grained analysis of decades (Figure 4B) shows some interesting findings. For solid cancers, cumulative incidences of 12-month risks gradually increased from 0.98% in the 1970s to 2.1% in the 2010s and 2.9% in the 2020s cohort (*p* < 0.0001, Gray’s test). Furthermore, survival risks for cardiovascular COD and hematological cancers reduced in the same time span (3.1% to 1.6%, and 4.0% and 2.1%, respectively).

## 4. Discussion

SEER data can be used in many different study designs and analysis settings. Within this work, we applied, for the first time, a competing risk analysis, estimating yearly subdistribution hazard ratios to analyze AML survival from epidemiological registry data. We identified a general and continuous trend of improving survival in AML patients throughout the 46 years that were analyzed. The data shows how clinical research ultimately leads to improvement in the target population. The application of a fixed reference year allowed us to visualize historic trends on a fine-grained level. Under the assumption of proportional hazards between the reference year 1975 and each analyzed following year, the estimated yearly sHRs are characterizing the medical progress in AML treatment and care, as well as other influencing factors. Overall survival is influenced by various factors that can ultimately lead to shifts in the observed data that we analyzed. Four major domains contribute to the survival estimand: (i) disease specific characteristics (leukemic pace, severity (e.g., influenced by genetic set-up), the epidemiologic profile of the patient cohort (e.g., mean age), available diagnosis (lead-time bias)); (ii) treatment factors (approval, effectiveness, availability, knowledge on therapies); (iii) supportive care and follow-up care (i.e., prevention or management of late- or long-term effects); (iv) public-health factors (general life expectancy, nutritional status, other hazards (natural, environmental, occupational, etc.)). Our estimates of survival can and will be influenced by changes in one or many of these domains. The benefit of the CRA is that it can more clearly attribute the general trend to the improvement of AML-related mortality to advances and access to treatments. The gradual improvement in sHRs indicates that many influencing factors are acting over time, e.g., continuous improvement of access to (new) therapeutic options, to improved supportive care and follow-up care, health literacy, potential faster and more precise diagnostic workup, and further unobserved factors. For the interpretation of results, it is important to keep in mind that hazard ratios, in general, do not allow assessment of the absolute risk for mortality. Thus, the presented HRs (either from the Cox-model or the sub-distribution sHRs [CRA]) should be interpreted in combination with the estimated survival probabilities.

Subgroup analysis: Our analysis of subgroups by age, sex and treatment status showed, that differential trends could be identified only for age and treatment status. The latter is an obvious finding, i.e., patients not undergoing chemotherapy, as major treatment option, exhibit generally the same low survival chances over the last 40 decades. Reasons for not treating a patient with chemotherapy may be various and influenced by patient fitness, eligibility, willingness, or late diagnoses. More generally, factors for treatment decision making (oncologist and patient related) were reported by Loh et al. [24] Additionally, e.g., Genç et al. identified access to blood bank support, availability of chemotherapeutics and targeted therapies, as well as financial aspects as major barriers in AML treatment [25]. These considerations gain even more importance, when discussed in the context of low- and middle-income countries [26]. More detailed insights of the elderly “no-treatment” subgroup of the SEER data was recently published [27]. With respect to age, the most striking finding was the delayed onset of a trend in the early 2000s for patients older than 60 years at diagnosis. This subgroup showed almost no progress in the decades before. We hypothesized that this is very likely caused by a change in treatment policy. Unfortunately, no directly available data could be used to perform more detailed analyses, here. Historically, curative treatment was primarily limited to younger patients, largely due to the intense nature of the procedures and the associated risks which were considered too substantial for older patients [28]. Oran and Weisdorf found a strong association of less leukemia therapy and older age with increasing comorbidities [29]. Hubscher et al. performed a meta-analysis of 24 studies and described a decline in the proportion of elderly AML patients receiving no active antileukemic treatment, on average, from ca 60% to 70% in 1999 to around 50% in 2016 [30]. Medeiros reported comparable numbers for the period between 2000 and 2009 [31]. Over the past decades, the major advancement for older patients was the approval of hypomethylating therapies, such as azacytidine and decitabine [32,33,34,35,36]. Also, low-dose cytarabine-based combination chemotherapies became available [37,38,39]. Due to emerging data favoring less intensive treatment in elderly patients [40,41], we hypothesized a switch of elderly patients receiving low intensive HMA-containing therapies instead of both intensive induction treatment and no antileukemic treatment. For a profound discussion of this data see [28]. Additionally, data shows an increase in the percentage of older patients with AML undergoing allogeneic stem cell transplantation [42,43] as well as improved relapse rates [44] and improved survival [45] after transplantation in older patients. Passweg et al., recently reported updated statistics on allogeneic HCT in Europe based on EBMT data [46]. Overall, the increase in older patients receiving active anti-leukemic treatment in combination with new therapy options should be viewed in combination with the identified delayed onset of an improvement in survival.

Competing events: The analysis for the composite event “death of other reasons” (shown in the Supplementary Figure S2) is impacted by patient’s survival related to late and long-term effects of AML and AML therapy. Confidence intervals for the estimated sub-distribution HRs were too wide to allow a more detailed and profound interpretation of trends based on the annual estimates. Though, the comparison of 12-month cumulative incidences by decade for these competing events indicated that trends exist. Particularly, incidence of cardiovascular death and subsequent hematological cancers decreased, while risk for death after a subsequent solid cancer increased in recent decades. Here, a more complex situation takes effect. Since treatment advancements for these late effects occur in the later decades, these AML patients benefit from extended survival, e.g., not dying from a myocardial disease, but thus have an increased chance to live to another cancer disease. As additional factors, which may contribute to this development, advances in diagnostic imaging, genetic profiling, availability of liquid biopsy and other diagnostic options have to be named, resulting in an increased probability to detect cancer. Given the SEER data, we found a shift in loci of solid cancers over the analyzed decades. Especially, colon cancers, as reasons for death after AML, reduced from 10% to 5% between the 1970s and the 2020s. Similarly, lung and bronchus cancers reduced from 21% to 14%. The group of miscellaneous malignant cancers increased to over 40% since the 2000s. It is unclear whether this is specific to AML patients or a general trend occurring in our dataset. Nevertheless, the prognostic properties of these malignant diseases might contribute to the changes in survival risk described above.

Early death rate: The (slight) decline in the early death rate can be explained by timing of diagnosis, toxicity of treatment, composition of patient cohorts (decreased proportion of frail patients in intensive treatment regimens over time), or combinations of those factors. None of these potentially related factors could be analyzed in this dataset. Also, the shift in reasons for early death could not be analyzed in detail, given the data.

Limitations: The complete SEER data represents approximately 46% of the US population. The representativeness of the SEER data was analyzed before. In 2004, Merill and Dearden found that overall “cancer site-specific mortality rates derived from SEER data tend to under-represent the US cancer mortality experience for white males and females and black males”, especially for tobacco-related cancers [47]. Their analyses of leukemias (diagnosed between 1992 and 2000) showed an acceptable overall rate ratio between total US and SEER numbers of 0.98 for black females, 1.02 for black males, 1.01 for white females, and 1.01 for white males. Additionally, this acceptable rate ratio was found across all analyzed years and age groups. This result encouraged the idea that the covered years show sufficient representativeness for our target population. In 2016, Kuo and Mobley reported no noticeable differences in SEER vs. non-SEER data sources for breast cancer and colorectal cancer cohorts [48]. Lu et al. recently performed a systematic analysis of the changes in SEER data using the population stability index (PSI) [49]. They concluded that “the U.S. cancer population after 2015 significantly differs from the population in 2000 in terms of sex, age, and cancer groups, but the differences between any two consecutive years are ignorable, aligning with previous epidemiology surveillance reports”. The authors pointed out that the PSI has limitations, e.g., it can only use categorical information leading to information loss due to binning of continuous variables. Also, their analysis compared selected years in an exemplary manner to analyze changes within the cohort, but the authors did not compare to a reference population.

While analyses of representativeness were usually made for the whole registry, the situation within the subset of AML patients is still unknown. Additionally, we described changes in the composition of population characteristics between decades in Supplementary Tables S6 and S7. These differences may affect the survival estimates to some extent. Also, the presented results should be interpreted in the context of the US health system and FDA approved treatment regimens. Though, in hematology, FDA approval of relevant new medication and substances is usually implemented in most developed countries without longer delay. Nevertheless, representativeness and transfer of our findings to other health systems should be checked in future studies. Further limitations comprise that treatment documentation can be biased in SEER data due to the completeness of the variables and the biases associated with unmeasured reasons for receiving or not receiving chemotherapy. Cause of death coding may be inexact, i.e., a misclassification might have happened. For example, a cardiac event coded as non-AML-related might actually result from AML-related cardiotoxicity. Physicians must decide whether to classify such events as AML-related or competing causes of death, and without autopsy confirmation, these classifications may be incorrect. The SEER documentation (https://seer.cancer.gov/causespecific/, accessed on 1 November 2025) recognizes also, that “in some cases, attribution of a single cause of death may be difficult and misattribution may occur. For example, a death may be attributed to the site of metastasis instead of the primary site”. Furthermore, evolving diagnostic capabilities—such as improved laboratory and imaging methods for detecting secondary cancers—may influence cause of death classification over time. For our analysis, we have to depend on the decision how to code the reason of death, based on SEER standards.

With respect to the quality of survival information the data contained 8251 cases with incomplete date information, such that the reported (by SEER pre-computed) survival times of these patients could, in principle, be different. For future studies, more detailed patient information is crucial to characterize trends is OS in more detail. Follow-up of the 2020s decade is short and likely be based on more severe cases, who died and could already be documented completely. With respect to ED rates, we identified similarities to published data from the same source. For further discussion, we would like to refer to the points made by Sasaki et al. [11].

Current and future expectations: While definitive data for the 2020–2021 cohort and following years are pending, there is a strong expectation that ongoing improvements will further enhance survival rates. This anticipated positive trajectory is driven by cutting-edge treatments like immunotherapies, enhanced genetic understanding guiding treatment choice, and ongoing clinical trials exploring novel therapeutic combinations. For our aim, which is to characterize trends in OS, KM and sub-distribution HR were well suited, while from a patient perspective relative survival might be the more relevant information. To our knowledge no combination of relative survival analysis and competing risks has been explored yet but it could be a relevant future direction of methodological research.

## 5. Conclusions

We provided a concise analysis of historic trends in survival after AML. The detected ongoing trend seems encouraging due to the improving survival rates, that are detected in clinical trials and are translated in practice and reach the target population. Our analysis also revealed barriers, like the delayed access of the older population to effective treatment. Overall, the methodological approach used can be applied widely, to elucidate trends in survival or other event rates within medicine.

## Figures and Tables

**Table 1:** Descriptive statistics of AML cases documented in the SEER dataset.

	N	Statistic
Sex, n(%)		
Female	17,095	(45.5%)
Male	20,520	(54.6%)
Age at diagnosis(y) mean(SD)	37,615	64.8 (17.2)
Chemotherapy, n(%)		
Yes	25,214	(67.0%)
No/unknown	12,401	(33.0%)
First malignant tumor, n(%)	28,340	(75.3%)
Race, n(%)		
Black	2225	(5.9%)
White	31,579	(83.9%)
Other (American Indian, Native, Pacific Islander)	3718	(9.9%)
Unknown	93	(0.3%)

Footnotes: SD: standard deviation, n: number of patients.

## Data Availability

The data presented in this study are available in the US national cancer institute (NCI) SEER Databases at https://seer.cancer.gov/data/, accessed on 1 November 2024.

## Supplementary Materials

The following supporting information can be downloaded at: https://www.mdpi.com/article/10.3390/cancers17233735/s1, Supplementary Text S1: Complete SEER citation information and extracted variables; Supplementary Text S2: Definition of the Cox model to estimate the Fine and Gray model in SAS; Supplementary Table S1: SEER recode of the Acute Myeloid Leukemias subgroup; Supplementary Table S2: Summary of state of documentation of survival in extracted SEER dataset; Supplementary Table S3: Redefined classes of causes of death and their contained ICD-O 3 2023 coded causes; Supplementary Table S4: Hazard ratios for the event “AML-related death” used to generate panel plots in Figure 3; Supplementary Table S5: Summary of the available sample sizes per year; Supplementary Table S6: Historic changes in the SEER data with respect to relevant variables; Supplementary Table S7: Characterization of patients treated with and without chemotherapy by decade; Supplementary Table S8: Percentage of early deaths and 95%CI by decade of diagnosis; Supplementary Table S9: Development of causes of death by decade of diagnosis. Absolute and relative frequencies; Supplementary Figure S1: Sensitivity analyses; Supplementary Figure S2: Complete CRA of whole AML cohort showing also the competing event (other reasons).

## Abbreviations

The following abbreviations are used in this manuscript:

AML	Acute myeloid leukemia
CRA	Competing risk analysis
COD	Cause of death
ED	Early death
FDA	U.S. Food and Drug Administration
HCT	Hematological cell transplantation
HR	Hazard ratio
KM	Kaplan–Meier
OS	Overall survival
PSI	Population stability index
SEER	Surveillance Epidemiology and End Results (SEER) Program
sHR	Hazard-ratio estimated from a sub-distribution Fine-Grey model
