# Supplementary material for: Characterizing Overall Survival of Patients with Acute Myeloid Leukemia: A Competing Risk Analysis of SEER Data Covering 46 Years
Source: Cancers (Basel). 2025 Nov 22;17(23):3735. doi: 10.3390/cancers17233735 (PMC12690983; doi:10.3390/cancers17233735)
Supplement: Supplementary file 1 [file cancers-17-03735-s001.zip › cancers-3910378_suppl_figures.pdf]

cancers

MDPI

Supplementary Material

Characterizing Overall Survival of Patients with Acute Myeloid Leukemia: A Competing Risk Analysis of SEER Data Covering 46 Years

(A) Hazard ratios (Cox-PH model) by year of diagnosis

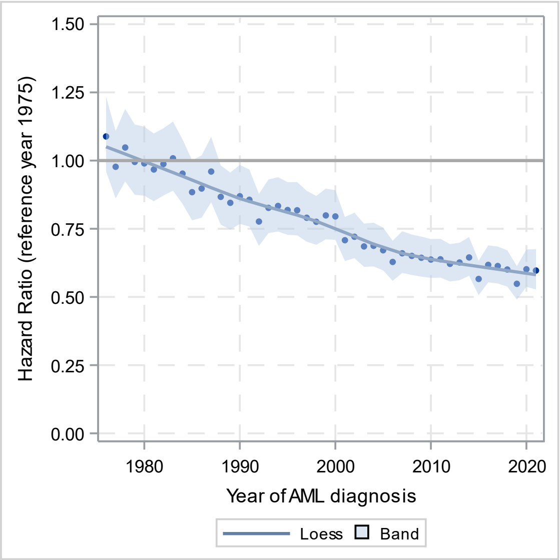

(B) Hazard ratios (Cox-PH model) by year of diagnosis by age group.

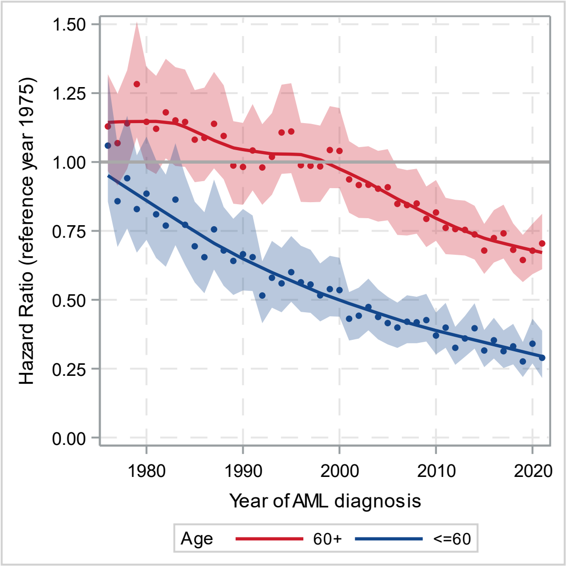

(C) Fine-Gray model (blue) without adjustment. Age- and sex-adjusted sub-distribution HR (red).

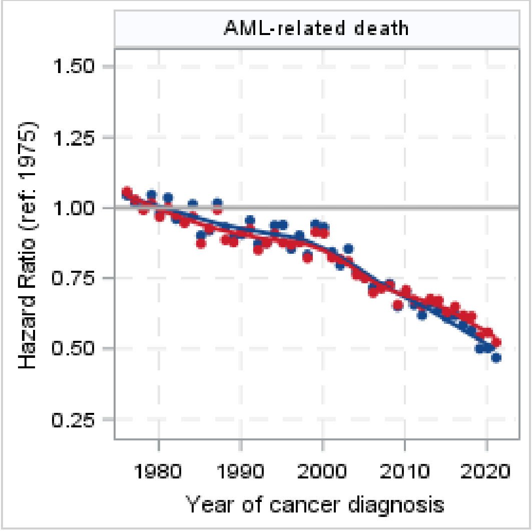

Figure S1. Sensitivity analyses.

 https://doi.org/10.3390/cancers17233735

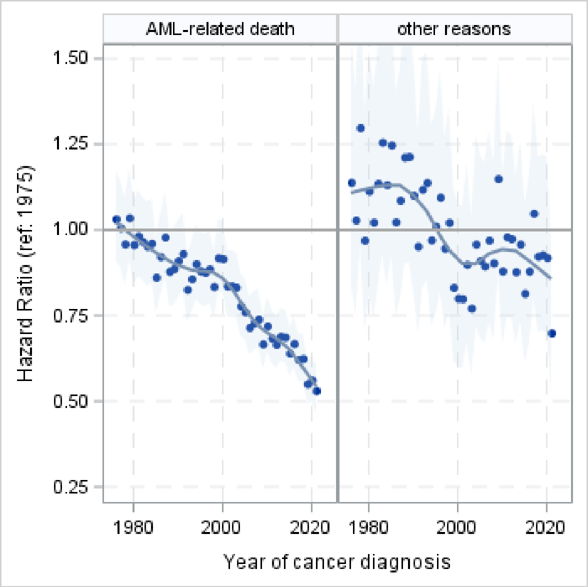

Figure S2. Complete CRA of whole AML cohort showing also the competing event (other reasons).
