# Supplementary material for: Characterizing Overall Survival of Patients with Acute Myeloid Leukemia: A Competing Risk Analysis of SEER Data Covering 46 Years
Source: Cancers (Basel). 2025 Nov 22;17(23):3735. doi: 10.3390/cancers17233735 (PMC12690983; doi:10.3390/cancers17233735)
Supplement: Supplementary file 1 [file cancers-17-03735-s001.zip › cancers-3910378_suppl_text_tables_proofread.pdf]

cancers

MDPI

Supplementary Material

Characterizing Overall Survival of Patients with Acute Myeloid Leukemia: A Competing Risk Analysis of SEER Data Covering 46 Years

Content

Text S1. Complete SEER citation information and extracted variables. 2

Text S2. Definition of the Cox model to estimate the Fine and Gray model in SAS. 2

Table S1. SEER recode of the Acute Myeloid Leukemias subgroup. 2

Table S2. Summary of state of documentation of survival in extracted SEER dataset. 3

Table S3. Redefined classes of causes of death and their contained ICD-O 3 2023 coded causes. 3

Table S4. Hazard ratios for the event "AML-related death" used to generate panel plots in Figure 3.. 4

Table S5. Summary of available sample sizes per year. 6

Table S6. Historic changes in the SEER data with respect to relevant variables. 7

Table S7. Characterization of patients treated with and without chemotherapy by decade. 7

Table S8. Percentage of early deaths and 95%CI by decade of diagnosis. 7

Table S9. Development of causes of death by decade of diagnosis. Absolute and relative frequencies. 8

GATHER checklist...8

Text S1. Complete SEER citation information and extracted variables.

Surveillance, Epidemiology, and End Results (SEER) Program (www.seer.cancer.gov) SEER*Stat Database: Incidence - SEER Research Data, 8 Registries, Nov 2023 Sub (1975-2021) - Linked To County Attributes - Time Dependent (1990-2022) Income/Rurality, 1969-2022 Counties.

Extracted variables: Age recode with <1 year olds, Race recode (White, Black, Other), Sex, Year of diagnosis, Site recode ICD-O-3/WHO 2008, Site recode ICD-O-3 2023 Revision, Site recode ICD-O-3 2023 Revision Expanded, Primary Site – labeled, ICCC site recode 3rd edition/IARC 2017, Site recode ICD-O-3/WHO 2008 (for SIRs), Diagnostic Confirmation, SEER historic stage A (1973-2015), Combined Summary Stage (2004+), Radiation recode, Chemotherapy recode (yes, no/unk), Time from diagnosis to treatment in days recode, COD to site recode, SEER cause-specific death classification, SEER other cause of death classification, Survival months, Survival months flag, COD to site rec KM, COD to site recode ICD-O-3 2023 Revision, COD to site recode ICD-O-3 2023 Revision Expanded (1999+), Total number of in situ/malignant tumors for patient, Total number of benign/borderline tumors for patient, First malignant primary indicator, Year of follow-up recode, Year of death recode, Patient ID, Age recode with single ages and 90+

Text S2. Definition of the Cox model to estimate the Fine and Gray model in SAS.

We used "eventcode" syntax in SAS *proc phreg* to estimate sub-distribution hazard ratios. Year of diagnosis was entered the model as fixed categorical factor. Contrasts were GLM coded with *ref=first*. Cofactors (e.g. sex) were added as categorical fixed factors and an interaction term with year of diagnosis was used. This allowed us to estimate yearly hazard ratios using the *hazardratio* statement in *proc phreg*. In all models "0" (alive) was used as censoring state, all other codes were considered competing to the eventcode.

Proportionality was assessed by visual inspection of the log(-log(CIF)) vs log(time) plots as estimated from the Fine-Gray models. 46 plots were generated to illustrate the pairwise comparison of the estimated CIF in the diagnosis year with the reference year. Here, selected plots are shown for the comparisons (1976 and 1977 vs 1975) as well as (1982, 1983, 2020 and 2021 vs 1975).

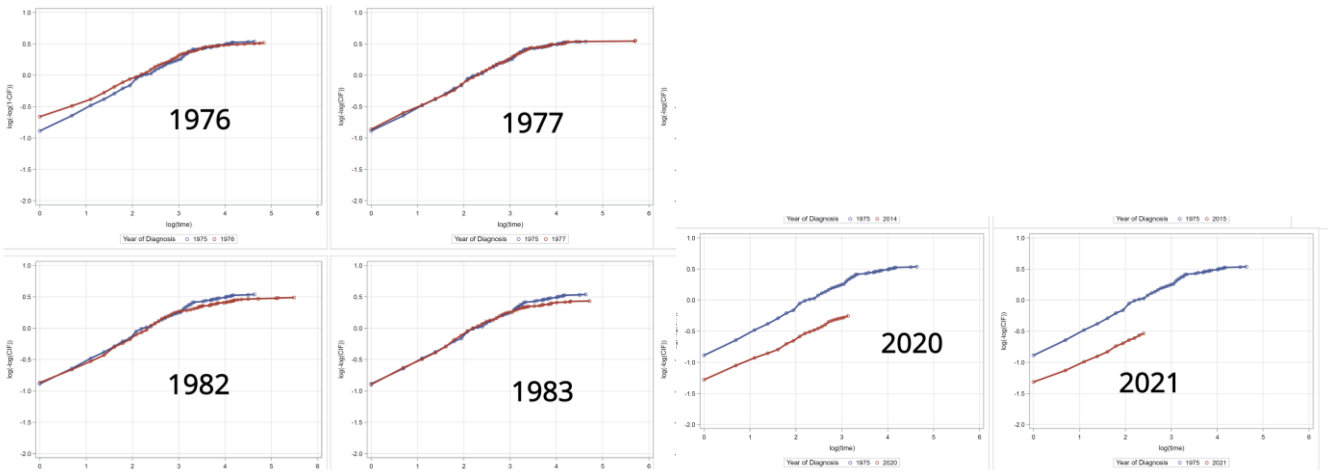

Table S1. SEER recode of the Acute Myeloid Leukemias subgroup.

	ICD-O-3 Site	ICD-O-3 Histology (Type)
**Acute Myeloid Leukemias**	C000-C809	9840, 9861, 9865-9867, 9869-9874, 9877-9879, 9891, 9895-9898, 9910-9912, 9920, 9930-9931, 9987

Based on https://seer.cancer.gov/siterecode/icdo3_2023_expanded/.

Table S2. Summary of state of documentation of survival in extracted SEER dataset.

Survival status code	Frequency	Percent
Complete dates available, 0 days survival	257	0.68
Complete dates available, >0 days survival	29,107	77.38
Incomplete dates available, cannot be zero days	6,928	18.42
Incomplete dates available, could be zero days	1,323	3.52

The two categories for "Incomplete dates ..” correspond to the quality assessment of the SEER data with respect to missing month or day in any relevant dates for computation of survival information. We used all precomputed survival times that were contained in the data regardless of this classification.

Table S3. Redefined classes of causes of death and their contained ICD-O 3 2023 coded causes.

Cause of death class for analysis	ICD-O 3 (2023) Recoded	Absolute frequency
Alive at last FU (censored)	Alive	5424
AML related death	Acute Myeloid Leukemias	20441
	Myeloproliferative Neoplasms and Myelodysplastic Syndromes	709
	Other Leukemias	5262
No COD coded	State DC not available or state DC available but no COD	296
Cardiovascular Disease	Cerebrovascular diseases	148
	Diseases of arteries, arterioles and capillaries	36
	Hypertensive disease	55
	Ischemic heart disease	498
	Other and unspecified disorders of the circulatory system	354
	Pulmonary heart disease and diseases of pulmonary circulation	28
Solid Cancer	Anus, Anal Canal and Anorectum	1
	Benign and Borderline: All Other sites	21
	Biliary Other	1
	Bones And Joints	1
	Brain (Malignant)	14
	Brain, CNS Other and Intracranial Gland (Benign and Borderline)	3
	Breast	80
	Cervix	1
	Colon And Rectum (Excluding Appendix)	54
	Corpus	23
	Digestive Other	3
	Esophagus	11
	Fallopian Tube	1
	Gallbladder	1
	Heart, Mediastinum And Pleura	1
	Intrahepatic Bile Duct	6
	Kidney Parenchyma	9
	Larynx	1
	Liver	17
	Lung And Bronchus	156
	Melanoma Of The Skin	10
	Miscellaneous Neoplasms	304
	Mouth Other	2
	Nasopharynx	1
	Oropharynx	1

	Other Non-Epithelial Skin	11
	Ovary	56
	Palate Excluding Soft And Uvula	1
	Pancreas	27
	Prostate	37
	Small Intestine	2
	Soft Tissue	14
	Stomach	9
	Stomach and Duodenal Ulcers	8
	Thymus	2
	Thyroid	3
	Tongue Anterior	5
	Urinary Bladder	19
	Vagina	1
	Vulva	1
Infection and Sepsis	COVID (2020+ only)	41
	Other infectious and Parasitic Diseases incl HIV	213
	Pneumonia and Influenza	201
	Septicemia	100
Non-cancer cond.	Alzheimers (ICD-9 and ICD-10 only)	14
	Certain Conditions Originating in Perinatal Period	1
	Chronic Liver Disease and Cirrhosis	47
	Chronic Obstructive Pulmonary Disease and Allied Conditions	122
	Complications of Pregnancy, Childbirth, Puerperium	13
	Complications of medical and surgical care (Y40-Y84, Y88) (ICD-10 only, 1999+)	35
	Congenital Anomalies	24
	Diabetes Mellitus	78
	Nephritis, Nephrotic Syndrome and Nephrosis	77
Haematological cancers	Chronic lymphocytic leukemia (CLL)/Small lymphocytic lymphoma	86
	Hodgkin Lymphomas	44
	Lymphoid Leukemias (excluding CLL/SLL)	52
	Miscellaneous Hematopoietic Neoplasms	15
	Myeloproliferative Neoplasms and Myelodysplastic Syndromes	373
	Non-Hodgkin Lymphomas	269
	Plasma Cell Neoplasms and Immunoproliferative Diseases	102
	Precursor Lymphoid Neoplasms	322
Other COD	Accidents and Adverse Effects	177
	Homicide and Legal Intervention	3
	Other COD	943
	Suicide and Self-Inflicted Injury	38
	Symptoms, Signs and Ill-Defined Conditions	55

Table S4. Hazard ratios for the event "AML-related death" used to generate panel plots in Figure 3. All data shows hazard ratios with common reference year 1975 and a pointwise 95%CI either for the whole cohort or by subgroup.

**Year of diagnosis**	**Whole cohort**	**Age <= 60**	**61+y**	**Chemotherapy**	**No therapy / unknown**	**Female**	**Male**
1975 (reference)	1	1	1	1	1	1	1
'76	1.033 (0.909; 1.174)	1.186 (0.977; 1.441)	0.995 (0.840; 1.180)	1.053 (0.905; 1.225)	0.998 (0.785; 1.270)	0.958 (0.798; 1.150)	1.107 (0.926; 1.324)
'77	1.007 (0.889; 1.141)	0.989 (0.809; 1.210)	1.060 (0.903; 1.245)	0.974 (0.839; 1.130)	1.064 (0.841; 1.345)	1.237 (0.787; 1.943)	1.061 (0.694; 1.621)
'78	0.960 (0.841; 1.095)	0.996 (0.812; 1.222)	0.998 (0.839; 1.186)	0.979 (0.839; 1.142)	0.925 (0.721; 1.187)	0.957 (0.799; 1.146)	1.056 (0.888; 1.257)
'79	1.036 (0.911; 1.179)	0.933 (0.769; 1.132)	1.144 (0.959; 1.366)	0.950 (0.818; 1.104)	1.216 (0.950; 1.557)	1.101 (0.694; 1.747)	0.971 (0.631; 1.493)

1980	0.958 (0.844; 1.088)	1.004 (0.828; 1.217)	0.974 (0.820; 1.157)	0.978 (0.846; 1.130)	0.921 (0.715; 1.187)	0.867 (0.716; 1.051)	1.047 (0.873; 1.256)
‘81	0.983 (0.866; 1.117)	0.887 (0.724; 1.087)	1.098 (0.932; 1.292)	0.942 (0.815; 1.090)	1.067 (0.830; 1.372)	1.462 (0.938; 2.278)	1.172 (0.775; 1.772)
‘82	0.967 (0.853; 1.096)	0.824 (0.675; 1.006)	1.077 (0.916; 1.266)	0.915 (0.790; 1.058)	1.064 (0.835; 1.355)	1.023 (0.851; 1.230)	1.050 (0.877; 1.256)
‘83	0.953 (0.838; 1.085)	0.881 (0.716; 1.083)	1.003 (0.848; 1.186)	0.870 (0.746; 1.013)	1.120 (0.879; 1.427)	0.976 (0.599; 1.591)	0.963 (0.621; 1.496)
‘84	0.962 (0.847; 1.092)	0.827 (0.673; 1.016)	1.097 (0.932; 1.293)	0.891 (0.768; 1.034)	1.139 (0.889; 1.460)	0.976 (0.812; 1.173)	0.950 (0.796; 1.133)
‘85	0.863 (0.758; 0.982)	0.769 (0.628; 0.942)	0.972 (0.820; 1.151)	0.837 (0.720; 0.974)	0.916 (0.712; 1.178)	1.041 (0.635; 1.706)	1.148 (0.762; 1.731)
‘86	0.923 (0.812; 1.049)	0.867 (0.702; 1.070)	0.961 (0.816; 1.133)	0.893 (0.771; 1.035)	0.986 (0.766; 1.271)	0.858 (0.711; 1.035)	1.113 (0.937; 1.323)
‘87	0.980 (0.863; 1.113)	0.884 (0.722; 1.082)	1.076 (0.914; 1.267)	0.865 (0.746; 1.004)	1.288 (1.007; 1.648)	1.368 (0.872; 2.147)	0.755 (0.474; 1.203)
‘88	0.880 (0.776; 0.998)	0.806 (0.664; 0.979)	0.972 (0.824; 1.148)	0.833 (0.722; 0.960)	1.007 (0.778; 1.303)	0.896 (0.744; 1.079)	1.029 (0.867; 1.221)
‘89	0.888 (0.782; 1.007)	0.743 (0.602; 0.917)	0.969 (0.824; 1.139)	0.790 (0.682; 0.916)	1.184 (0.925; 1.516)	1.274 (0.807; 2.013)	1.034 (0.687; 1.555)
1990	0.911 (0.805; 1.031)	0.776 (0.629; 0.958)	0.984 (0.843; 1.149)	0.847 (0.736; 0.975)	1.089 (0.846; 1.403)	0.963 (0.797; 1.163)	0.950 (0.796; 1.135)
‘91	0.932 (0.826; 1.052)	0.697 (0.567; 0.858)	1.113 (0.958; 1.294)	0.828 (0.720; 0.952)	1.247 (0.980; 1.587)	1.297 (0.821; 2.051)	1.216 (0.815; 1.814)
‘92	0.827 (0.731; 0.936)	0.636 (0.513; 0.789)	0.989 (0.851; 1.150)	0.761 (0.659; 0.880)	0.977 (0.771; 1.238)	0.937 (0.782; 1.122)	0.986 (0.824; 1.180)
‘93	0.858 (0.761; 0.969)	0.692 (0.565; 0.849)	0.995 (0.855; 1.158)	0.818 (0.711; 0.942)	0.938 (0.742; 1.186)	1.157 (0.738; 1.813)	1.112 (0.741; 1.668)
‘94	0.902 (0.800; 1.018)	0.664 (0.541; 0.815)	1.102 (0.949; 1.280)	0.805 (0.699; 0.927)	1.119 (0.888; 1.409)	0.854 (0.709; 1.028)	0.872 (0.727; 1.045)
‘95	0.881 (0.782; 0.993)	0.744 (0.616; 0.899)	1.030 (0.880; 1.205)	0.815 (0.710; 0.935)	1.044 (0.823; 1.324)	1.187 (0.763; 1.845)	1.306 (0.880; 1.940)
‘96	0.877 (0.777; 0.990)	0.672 (0.546; 0.826)	0.987 (0.847; 1.149)	0.784 (0.680; 0.903)	1.068 (0.848; 1.345)	0.989 (0.824; 1.188)	0.871 (0.728; 1.043)
‘97	0.887 (0.788; 0.999)	0.648 (0.528; 0.795)	1.054 (0.909; 1.222)	0.750 (0.652; 0.863)	1.293 (1.032; 1.621)	0.842 (0.513; 1.381)	1.170 (0.775; 1.766)
‘98	0.836 (0.743; 0.939)	0.586 (0.478; 0.717)	0.991 (0.857; 1.147)	0.732 (0.639; 0.839)	1.109 (0.884; 1.391)	0.953 (0.794; 1.143)	1.006 (0.842; 1.202)
‘99	0.920 (0.818; 1.033)	0.668 (0.548; 0.815)	1.122 (0.970; 1.297)	0.741 (0.645; 0.851)	1.409 (1.135; 1.750)	1.084 (0.687; 1.712)	1.091 (0.723; 1.645)
2000	0.917 (0.818; 1.028)	0.655 (0.539; 0.795)	1.118 (0.969; 1.290)	0.780 (0.682; 0.891)	1.305 (1.048; 1.626)	0.839 (0.700; 1.006)	0.919 (0.772; 1.094)
‘01	0.837 (0.746; 0.939)	0.550 (0.451; 0.671)	1.031 (0.894; 1.189)	0.679 (0.592; 0.779)	1.222 (0.988; 1.512)	1.349 (0.868; 2.095)	1.105 (0.736; 1.660)
‘02	0.837 (0.745; 0.942)	0.599 (0.488; 0.734)	0.958 (0.827; 1.110)	0.715 (0.622; 0.822)	1.124 (0.902; 1.400)	0.868 (0.722; 1.044)	0.908 (0.763; 1.080)
‘03	0.834 (0.743; 0.936)	0.570 (0.469; 0.693)	1.066 (0.922; 1.232)	0.653 (0.569; 0.749)	1.367 (1.103; 1.695)	1.177 (0.752; 1.842)	1.229 (0.834; 1.812)
‘04	0.779 (0.693; 0.876)	0.471 (0.383; 0.579)	1.002 (0.867; 1.159)	0.610 (0.530; 0.703)	1.226 (0.986; 1.523)	0.924 (0.771; 1.108)	0.907 (0.766; 1.074)
‘05	0.762 (0.677; 0.857)	0.488 (0.397; 0.599)	0.970 (0.838; 1.122)	0.652 (0.567; 0.749)	1.065 (0.850; 1.334)	1.002 (0.622; 1.613)	1.159 (0.780; 1.721)
‘06	0.716 (0.636; 0.806)	0.480 (0.392; 0.589)	0.894 (0.771; 1.036)	0.617 (0.538; 0.709)	1.016 (0.807; 1.278)	0.908 (0.766; 1.078)	0.955 (0.805; 1.133)
‘07	0.730 (0.649; 0.822)	0.447 (0.362; 0.552)	0.916 (0.792; 1.058)	0.576 (0.500; 0.662)	1.201 (0.965; 1.495)	0.901 (0.569; 1.427)	0.999 (0.665; 1.503)
‘08	0.740 (0.660; 0.830)	0.475 (0.390; 0.579)	0.932 (0.807; 1.075)	0.601 (0.525; 0.687)	1.156 (0.929; 1.439)	0.794 (0.664; 0.950)	0.858 (0.724; 1.018)
‘09	0.668 (0.595; 0.750)	0.450 (0.368; 0.551)	0.796 (0.688; 0.920)	0.561 (0.489; 0.643)	0.960 (0.770; 1.198)	1.330 (0.866; 2.043)	0.953 (0.631; 1.437)

2010	0.721 (0.644; 0.806)	0.398 (0.324; 0.490)	0.916 (0.799; 1.050)	0.606 (0.531; 0.691)	1.052 (0.849; 1.303)	0.866 (0.730; 1.026)	0.854 (0.719; 1.013)
'11	0.685 (0.612; 0.767)	0.451 (0.368; 0.553)	0.790 (0.687; 0.908)	0.560 (0.490; 0.640)	1.061 (0.856; 1.316)	1.036 (0.666; 1.611)	1.212 (0.829; 1.773)
'12	0.667 (0.596; 0.745)	0.339 (0.272; 0.423)	0.795 (0.694; 0.910)	0.559 (0.491; 0.637)	1.080 (0.868; 1.344)	0.810 (0.677; 0.969)	0.993 (0.843; 1.168)
'13	0.691 (0.618; 0.772)	0.385 (0.312; 0.475)	0.824 (0.719; 0.945)	0.584 (0.513; 0.664)	1.144 (0.918; 1.426)	1.113 (0.721; 1.720)	0.859 (0.571; 1.292)
'14	0.688 (0.615; 0.769)	0.415 (0.337; 0.511)	0.781 (0.681; 0.896)	0.581 (0.510; 0.662)	1.064 (0.856; 1.323)	0.904 (0.763; 1.072)	0.861 (0.727; 1.019)
'15	0.641 (0.573; 0.718)	0.354 (0.286; 0.439)	0.767 (0.669; 0.880)	0.547 (0.481; 0.623)	1.169 (0.929; 1.471)	0.994 (0.643; 1.538)	1.025 (0.693; 1.516)
'16	0.669 (0.599; 0.747)	0.384 (0.311; 0.473)	0.761 (0.664; 0.872)	0.564 (0.496; 0.641)	1.152 (0.923; 1.437)	0.829 (0.697; 0.986)	0.923 (0.779; 1.094)
'17	0.622 (0.556; 0.697)	0.328 (0.263; 0.409)	0.739 (0.644; 0.849)	0.515 (0.451; 0.588)	1.045 (0.837; 1.305)	1.192 (0.775; 1.833)	1.019 (0.687; 1.510)
'18	0.625 (0.559; 0.700)	0.317 (0.251; 0.400)	0.718 (0.626; 0.823)	0.544 (0.477; 0.619)	0.960 (0.766; 1.202)	0.844 (0.710; 1.004)	0.926 (0.786; 1.091)
'19	0.551 (0.491; 0.619)	0.271 (0.213; 0.344)	0.642 (0.558; 0.738)	0.453 (0.396; 0.518)	1.033 (0.820; 1.301)	0.928 (0.589; 1.462)	0.946 (0.643; 1.391)
2020	0.563 (0.500; 0.634)	0.283 (0.219; 0.367)	0.638 (0.553; 0.736)	0.467 (0.406; 0.537)	0.929 (0.737; 1.170)	0.771 (0.650; 0.914)	0.896 (0.763; 1.052)
'21	0.532 (0.467; 0.605)	0.276 (0.203; 0.375)	0.586 (0.503; 0.684)	0.383 (0.326; 0.451)	0.976 (0.774; 1.230)	1.043 (0.679; 1.601)	1.001 (0.684; 1.466)

Table S5. Summary of available sample sizes per year.

Year of diagnosis	Frequency	Cumulative frequency
1975	477	477
1976	508	985
1977	499	1484
1978	490	1974
1979	455	2429
1980	481	2910
1981	473	3383
1982	525	3908
1983	514	4422
1984	547	4969
1985	550	5519
1986	499	6018
1987	533	6551
1988	535	7086
1989	563	7649
1990	541	8190
1991	605	8795
1992	602	9397
1993	686	10083
1994	675	10758
1995	721	11479
1996	674	12153
1997	720	12873
1998	819	13692
1999	783	14475
2000	858	15333

2001	898	16231
2002	838	17069
2003	861	17930
2004	796	18726
2005	815	19541
2006	825	20366
2007	861	21227
2008	944	22171
2009	956	23127
2010	1066	24193
2011	1099	25292
2012	1186	26478
2013	1221	27699
2014	1190	28889
2015	1154	30043
2016	1260	31303
2017	1236	32539
2018	1246	33785
2019	1279	35064
2020	1249	36313
2021	1302	37615

Table S6. Historic changes in the SEER data with respect to relevant variables.

	Decade of diagnosis						
	1975-1979	1980-1989	1990-1999	2000-2009	2010-2019	2020-2021	p-value
Chemotherapy, %	61·1%	63·9%	64·0%	64·4%	71·9%	73·2%	<·0001a
Female sex, %	47·0%	45·7%	45·8%	46·0%	44·5%	45·0%	0·1472a
First malignant tumour, %	90·8%	88·5%	83·7%	79·2%	62·0%	60·3%	<·0001a
Ethnicity, %							<·0001a
White	91·2%	87·9%	86·6%	80·7%	79·3%	84·0%	
Black	4·0%	4·9%	4·7%	5·9%	7·2%	7·3%	
Other	4·7%	7·1%	8·7%	10·6%	11·7%	12·9%	
Age, mean (SD)	63·12 (17·81)	63·22 (17·77)	63·77 (17·56)	64·44 (17·49)	66·20 (16·41)	67·37 (15·88)	<·0001b
Footnotes: a – Chi-square test, b – ANOVA; SD: standard deviation							

Table S7. Characterization of patients treated with and without chemotherapy by decade.

	**1970′s**		**1980′s**		**1990′s**		**2000′s**		**2010′s**		**2020′s**	
	**Chemo**	**No chemo (+un-known)**	**Chemo**	**No chemo (+un-known)**	**Chemo**	**No chemo (+un-known)**	**Chemo**	**No chemo (+un-known)**	**Chemo**	**No chemo (+un-known)**	**Chemo**	**No chemo (+un-known)**
Age,mean (SD)	57.4 (17.9)	72.1 (13.6)	57.3 (17.5)	73.7 (12.9)	58.4 (17.3)	73.4 (13.5)	58.6 (17.0)	75.0 (12.7)	62.3 (16.2)	76.2 (12.2)	64.2 (15.7)	75.9 (13.0)
Fe-male,n(%)	692 (60.6)	450 (39.4)	1485 (62.3)	900 (37.7)	2000 (64.0)	1125 (36.0)	2522 (63.4)	1458 (36.7)	3753 (70.6)	1563 (29.4)	824 (71.8)	323 (28.2)
Male, n(%)	792 (61.5)	495 (38.5)	1849 (65.2)	986 (34.8)	2372 (64.0)	1329 (35.9)	3047 (65.2)	1625 (34.8)	4835 (73.0)	1769 (27.0)	1043 (74.3)	361 (25.7)

Please note that treatment information from the SEER program can be substantially biased, according to official information given by the SEER documentation.

Table S8. Percentage of early deaths and 95%CI by decade of diagnosis.

**ED time**	**1970's**	**1980's**	**1990's**	**2000's**	**2010's**	**2020's**	**p-value (Log rank test#)**

1-month ED rate	43.2% (41.3% to 45.2%)	39.9% (38.6% to 41.3 %)	37.4% (36.3% to 38.6%)	33.5% (32.5% to 34.5%)	29.3% (28.5% to 30.1%)	29.9% (28.2% to 31.8%)	<·0001
2-month ED rate	51.0% (49.0% to 53.0%)	46.9% (45.5% to 48.2 %)	44.0% (42.9% to 45.2%)	39.7% (38.6% to 40.7%)	35.0% (34.2% to 35.9%)	35.5% (33.6% to 37.4%)	<·0001

ED – early death;

Footnote: ED rates were estimated by Kaplan-Meier analysis reporting the 1-month and 2-month failure rate.

Log rank tests were computed in a modified Kaplan-Meier analysis where all events occurring later than the selected ED time were censored at the ED time.

Table S9. Development of causes of death by decade of diagnosis. Absolute and relative frequencies.

Cause of death, N(%) #	Decade of Diagnosis							
	**1975-1979**	**1980-1989**	**1990-1999**	**2000-2009**	**2010-2019**	**2020-2021**	**Total**	**Test†**
AML-related death	1976 (82·0)	4110 (80·8)	5270 (82·6)	6268 (83·3)	7707 (81·8)	1081(79·4)	26412	0·0011
Other causes of death								
Cardiovascular death	111 (4·6)	218 (4·3)	239 (3·7)	255 (3·4)	257(2·7)	39 (2·9)	1119	<·0001
Solid cancer death	38 (1·6)	90 (1·8)	132 (2·1)	238 (3·2)	356 (3·8)	65 (4·8)	919	<·0001
Infection and sepsis	37 (1·5)	103 (2·0)	102 (1·6)	109 (1·5)	180 (1·9)	24 (1·8)	555	0·1048
Non-cancer condition	17 (0·7)	64 (1·3)	103 (1·6)	103 (1·4)	113 (1·2)	11 (0·8)	411	0·0108
Haematological cancer	125 (5·2)	243 (4·8)	232 (3·6)	255 (3·4)	363 (3·9)	45 (3·3)	1263	<·0001
Other COD	74 (3·1)	198 (3·9)	265 (4·2)	255 (3·4)	363 (3·9)	61 (4·5)	1216	0·0502
Total deaths	2409	5084	6383	7529	9425	1361	32191	

# Percentages were computed within decades.

† Logistic regression model testing for a change in probability over decades. Tested against the pooled cohort of all other CODs.

GATHER checklist

Item #	Checklist Item	Reported on page #
	**Objectives and funding**	
1	Define the indicator(s), populations (including age, sex, and geographic entities), and time period(s) for which estimates were made.	p.6
2	List the funding sources for the work.	Not applicable
	**Data inputs**	
	For all data inputs from multiple sources that are synthesized as part of the study:	
3	Describe how the data were identified and how the data were accessed	p.6
4	Specify the inclusion and exclusion criteria. Identify all ad-hoc exclusions	p.6
5	Provide information on all included data sources and their main characteristics. For each data source used, report reference information or contact name/institution, population represented, data collection method, year(s) of data collection, sex and age range, diagnostic criteria or measurement method, and sample size, as relevant	p.6
6	Identify and describe any categories of input data that have potentially important biases (e.g., based on characteristics listed in item 5)	Method section
	For data inputs that contribute to the analysis but were not synthesized as part of the study:	
7	Describe and give sources for any other data inputs.	Not applicable
	For all data inputs:	
8	Provide all data inputs in a file format from which data can be efficiently extracted (e.g., a spreadsheet rather than a PDF), including all relevant meta-data listed in item 5. For any data inputs that cannot be shared because of ethical or legal reasons, such as third-party ownership, provide a contact name or the name of the institution that retains the right to the data.	No sharing possible
	**Data analysis**	
9	Provide a conceptual overview of the data analysis method. A diagram may be helpful.	Methods section
10	Provide a detailed description of all steps of the analysis, including mathematical formulae. This description should cover, as relevant, data cleaning, data pre-processing, data adjustments and weighting of data sources, and mathematical or statistical model(s).	Methods section

11	Describe how candidate models were evaluated and how the final model(s) were selected.	Methods section
12	Provide the results of an evaluation of model performance, if done, as well as the results of any relevant sensitivity analysis.	Supplementary material
13	Describe methods for calculating uncertainty of the estimates. State which sources of uncertainty were, and were not, accounted for in the uncertainty analysis.	Methods section
14	State how analytic or statistical source code used to generate estimates can be accessed.	On request, email to corresponding author
	**Results and Discussion**	
15	Provide published estimates in a file format from which data can be efficiently extracted	Table S4
16	Report a quantitative measure of the uncertainty of the estimates (e.g., uncertainty intervals).	95%CI were reported with all HRs
17	Interpret results in light of existing evidence. If updating a previous set of estimates, describe the reasons for changes in estimates.	Discussion section, p15
18	Discuss limitations of the estimates. Include a discussion of any modelling assumptions or data limitations that affect interpretation of the estimates.	p.17, p.18

Note: Page numbers refer to the submitted manuscript and may be different in the published version of the article.
